# Artificial Neurons and Synapses Based on Al/a-SiN_x_O_y_:H/P^+^-Si Device with Tunable Resistive Switching from Threshold to Memory

**DOI:** 10.3390/nano12030311

**Published:** 2022-01-18

**Authors:** Kangmin Leng, Xu Zhu, Zhongyuan Ma, Xinyue Yu, Jun Xu, Ling Xu, Wei Li, Kunji Chen

**Affiliations:** 1 The School of Electronic Science and Engineering, Nanjing University, Nanjing 210093, China; lengkangmin@foxmail.com (K.L.); zhuxu1998@hotmail.com (X.Z.); yuxinyue2083@126.com (X.Y.); junxu@nju.edu.cn (J.X.); xuling@nju.edu.cn (L.X.); weili@nju.edu.cn (W.L.); kjchen@nju.edu.cn (K.C.); 2 Collaborative Innovation Center of Advanced Microstructures, Nanjing University, Nanjing 210093, China; 3 Jiangsu Provincial Key Laboratory of Photonic and Electronic Materials Sciences and Technology, Nanjing University, Nanjing 210093, China

**Keywords:** brain-inspired computing, resistive switching, memory switching, threshold switching

## Abstract

As the building block of brain-inspired computing, resistive switching memory devices have recently attracted great interest due to their biological function to mimic synapses and neurons, which displays the memory switching or threshold switching characteristic. To make it possible for the Si-based artificial neurons and synapse to be integrated with the neuromorphic chip, the tunable threshold and memory switching characteristic is highly in demand for their perfect compatibility with the mature CMOS technology. We first report artificial neurons and synapses based on the Al/a-SiN_x_O_y_:H/P^+^-Si device with the tunable switching from threshold to memory can be realized by controlling the compliance current. It is found that volatile TS from Al/a-SiN_x_O_y_:H/P^+^-Si device under the lower compliance current is induced by the weak Si dangling bond conductive pathway, which originates from the broken Si-H bonds. While stable nonvolatile MS under the higher compliance current is attributed to the strong Si dangling bond conductive pathway, which is formed by the broken Si-H and Si-O bonds. Theoretical calculation reveals that the conduction mechanism of TS and MS agree with P-F model, space charge limited current model and Ohm’s law, respectively. The tunable TS and MS characteristic of Al/a-SiN_x_O_y_:H/P^+^-Si device can be successfully employed to mimic the biological behavior of neurons and synapse including the integrate-and-fire function, paired-pulse facilitation, long-term potentiation and long-term depression as well as spike-timing-dependent plasticity. Our discovery supplies an effective way to construct the neuromorphic devices for brain-inspired computing in the AI period.

## 1. Introduction

With the big data and artificial intelligence time approaching, brain-inspired computing is urgently needed to deal with the massive and diverse data. As the building block of brain-inspired computing, resistive switching memory (RSM) devices have recently attracted great interest due to their bioelectronic function to mimic synapses and neurons, which displays the memory switching (MS) or threshold switching (TS) characteristic [1,2,3,4]. To make it possible for the Si-based artificial neurons and synapse to be integrated with neuromorphic chip, the controllable MS and TS characteristic is in high demanded for their perfect compatibility with the mature CMOS technology [5]. In a hardware neural network, artificial electronic synapses modulate the signal transmission via the synaptic weight update, represented by the device conductance modification [6,7,8,9,10,11,12,13]. To ensure the bioelectronic synapse matrix works efficiently, TS memory is needed to emulate integrate-and-fire function of neurons, which is combined with MS to form the two fundamental elements for hardware neural networks [14,15,16,17]. In the long term, the realization of controllable MS and TS characteristics in the same Si-based resistive switching system remains a great challenge, which can guarantee the lower cost for fabrication of the neuromorphic chip [18,19,20]. Until now, the material scope of TS memory was mainly limited to oxide-based material such as HfO_2_ [18,21], Al_2_O_3_ [22], NiO [23,24], which dependents on the metal conductive pathway. According to the references [8,9], the precise control of ion migration in the resistive switching devices is the performance selection criteria for neuromorphic applications. However, the realization of resistive switching from TS to MS by tuning the Si dangling bond conductive pathway in Si-based RRAM devices is less reported [25,26,27,28,29,30].

In this article, we first report the observation of tunable switching from TS to MS in the Al/a-SiN_x_O_y_:H/P^+^-Si RSM with the compliance current increasing from 0.1 µA to 100 µA, displaying the reliable retention and long endurance. The transition from TS to MS is attributed to the evolution of the Si dangling bond pathway based on the analysis of FTIR, ESR and temperature dependent I-V characteristic. It is found that volatile TS from Al/a-SiN_x_O_y_:H/P^+^-Si device under the lower compliance current is induced by the weak Si dangling bond conductive pathway, which originates from the broken Si–H bonds, while stable nonvolatile MS under the higher compliance current is attributed to the strong Si dangling bond conductive pathway, which is formed by the broken Si–H and Si–O bonds. Theoretical calculation reveals that the conduction mechanism of TS and MS agree with P-F, SCLC model and Ohm’s law, respectively. The controllable TS and MS characteristic of Al/a-SiN_x_O_y_:H/P^+^-Si device can be successfully employed to mimic the biological behavior of neurons and synapse including the integrate-and-fire function, paired-pulse facilitation, long-term potentiation and long-term depression as well as spike-timing-dependent plasticity. Our discovery demonstrates a simple yet valid method to build artificial neurons and synapses with TS and MS characteristics, which present the potential application for neuromorphic computing.

## 2. Materials and Methods

The a-SiN_x_O_y_:H films were fabricated in a plasma-enhanced chemical vapor deposition system at 250 °C, with the P^+^ Si wafer as the substrate. The chemical reaction gases are SiH_4_, NH_3_ and N_2_O. The gas flow ratio of SiH_4_ to NH_3_ and N_2_O is 1:40:5. The aluminum top electrodes were grown on the surfaces of the a-SiN_x_O_y_:H films by thermal evaporation. A shadow mask was used to get circular electrode. For better contact, the back electrode was obtained by deposition of aluminum at the back side of the silicon substrate. The atomic concentration ratios of the a-SiN_x_O_y_:H films were obtained through XPS test using the PHI 5000 Versa Probe. The microstructures of the Al/a-SiN_x_O_y_:H/P^+^-Si device was analyzed using high-resolution cross-section transmission electron microscopy (HRTEM) with a JEOL 2100F electron microscope (JEOL. Akishima, Tokyo, Japan) operated at 200 kV. To demonstrate the paramagnetic center in the a-SiN_x_O_y_:H film at the pristine state, we carried out the ESR spectrum measurement, which was operated in the Bruker EMX-10/12 system (Bruker, Billerica, MA, USA) within a temperature range of 77–293 K. The Fourier Transform infrared spectroscopy (FTIR) was measured in the NEXUS870 system (Thermo Fisher, Waltham, MA, USA) to analyze the atomic bonding configuration in the a-SiN_x_O_y_:H films. The Agilent B1500A semiconductor analyzer (Agilent, Santa Clara, CA, USA) was used to explore the electrical behaviors of the devices in atmosphere. Additionally, the Lake Shore CRX-4K system (Lakeshore, Columbus, OH, USA) was adopted to check the temperature dependent I-V performances.

## 3. Results

**Figure 1:**
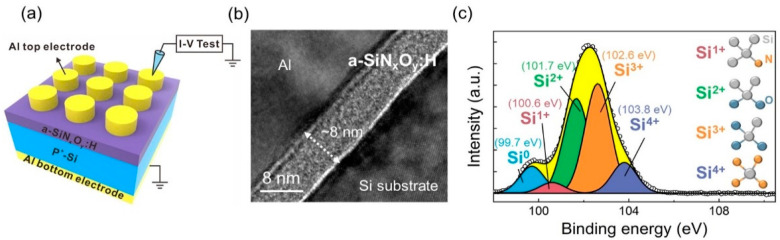
(**a**) Schematic illustration of the Al/a-SiN_x_O_y_:H/P^+^-Si RRAM device with electrical measurement; (**b**) Cross-sectional HRTEM image of the Al/a-SiN_x_O_y_:H/P^+^-Si RRAM device; (**c**) XPS spectrum of the as-deposited a-SiN_x_O_y_:H film.

The schematic diagram of Al/a-SiN_x_O_y_:H/P^+^-Si RSM device is shown in Figure 1a. An 8-nm-thick a-SiN_x_O_y_:H film was deposited on a highly doped Si substrate, which is served as the bottom electrode (BE) to ensure a good conductivity and flatness. Additionally, an aluminum (Al) layer was deposited on the surface of the a-SiN_x_O_y_:H layer as the top electrode (TE). A cross-sectional high-resolution transmission electron microscopy (HRTEM) image of the Al/a-SiN_x_O_y_:H/P^+^-Si RSM device is displayed in Figure 1b. It is observed that a-SiN_x_O_y_:H films of 8 nm is inserted between the Si substrate and Al electrode. The interfaces of the Si substrate, the a-SiN_x_O_y_:H films and the Al electrode are abrupt. The XPS peaks of the a-SiN_x_O_y_:H film corresponding to Si 2p is depicted in Figure 1c. The narrow XPS spectra can be deconvoluted into five peaks including Si^0^ (99.7 eV), Si^1+^ (100.6 eV), Si^2+^ (101.7 eV), Si^3+^ (102.6 eV) as well as Si^4+^ (103.8 eV) by Gaussian fittings. The five peaks correspond to Si, Si-Si_3_N, Si-Si_2_O_2_, Si-SiO_3_, and Si-N_4_ tetrahedral phases in a-SiN_x_O_y_:H films, respectively. As displayed in Figure 1c, the integration of the areas of the four sub-peaks shows that the area percentage of Si^0^, Si^1+^, Si^2+^, Si^3+^ and Si^4+^ are 9.8%, 3.8%, 34.9%, 40.4% and 11.1%, respectively [31]. The integration percentage of Si^0^ indicates that excess unreacted Si exist in the as-deposited a-SiN_x_O_y_:H films. The total integration percentage of silicon oxide specie reaches 75.3%, which is dominant in the a-SiN_x_O_y_:H films. The atomic percentage of Si, N and O is 46.1%, 30.1% and 19.4%, respectively.

**Figure 2:**
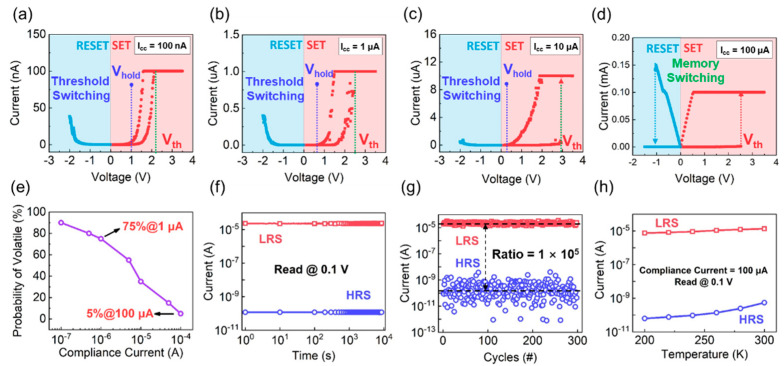
Threshold switching characteristic of the Al/a-SiN_x_O_y_:H/P^+^-Si device in the set process with I_cc_ of (**a**) 0.1 µA (**b**) 1 µA and (**c**) 10 µA, respectively; (**d**) Memory switching characteristic of the Al/a-SiN_x_O_y_:H/P^+^-Si device in the set process with I_cc_ of 100 µA; (**e**) Statistical probability of TS from the a-SiN_x_O_y_:H device with different I_cc_; (**f**) Retention characteristics of the Al/a-SiN_x_O_y_:H/P^+^-Si device at room temperature; (**g**) The endurance characteristic of the Al/a-SiN_x_O_y_:H/P^+^-Si device after 300 cycles under DC sweeping mode; (**h**) Temperature dependent I–V of the Al/a-SiN_x_O_y_:H/P^+^-Si device in HRS and LRS with I_cc_ of 100 µA.

As shown in Figure 2a–c, the evolution from TS to MS was observed from the Al/a-SiN_x_O_y_:H/P^+^-Si device with the I_cc_ (compliance current) increasing from 0.1 µA to 100 µA. With the I_cc_ ranging from 0.1µA to 10 µA, the Al/a-SiN_x_O_y_:H/P^+^-Si device switches from HRS to LRS when the positive sweeping voltage reaches a SET threshold voltage as marked by the green dashed line. The SET threshold voltages of 2.2 V, 2.5 V, and 3 V correspond to the I_cc_ of 0.1 µA, 1 µA and 10 µA. It indicates that the intensity of electric field becomes stronger with the increasing of I_cc_. Then, the device automatically switches back to the HRS when the sweeping voltage was lower than the holding voltage, which is marked as the blue dashed line. Interestingly, the holding voltage increases with the decreasing of I_cc_. The value of the holding voltage is 0.25 V, 0.6 V and 1 V. They are corresponding to 10 µA, 1 µA and 0.1 µA. When the I_cc_ increases to 100 µA, the Al/a-SiN_x_O_y_:H/P^+^-Si device presents MS behavior as shown in Figure 2d. Under the positive bias, the resistive switching from HRS to LRS is observed, and the LRS can be maintained for a long time after removing the voltage, which shows a typical feature of non-volatile switching. Under the negative bias, the resistive switching from LRS to HRS is detected, demonstrating a bipolar switching characteristic. It exhibits a large memory window of 1 × 10^5^. Figure 2e shows the statistical probability of TS from the Al/a-SiN_x_O_y_:H/P^+^-Si device under different I_cc_. With I_cc_ decreasing from 10^−4^ to 10^−7^, the probability of volatile switching is significantly increased. As for our device, the statistical probability of TS is near 100% when the I_cc_ is reduced to 0.1 µA. With I_cc_ increasing to 100 µA, the statistical probability of TS is reduced to 5%, which reveals that the switching between MS and TS can be realized by tuning the value of I_cc_. As shown in Figure 2f, the stable HRS and LRS of the Al/a-SiN_x_O_y_:H/P^+^-Si device can be maintained for 10^4^ s at a reading voltage of 0.1 V, displaying the characteristic of good retention. Moreover, the reproducible performance with memory window of 1 × 10^5^ can be detected after 300 cycles as presented in Figure 2g. To identify the properties of the conductive pathway in Al/a-SiN_x_O_y_:H/P^+^-Si device, we measured the temperature dependent I–V of HRS and LRS as displayed in Figure 2h. The current of HRS and LRS is enhanced with the temperature increasing from 200 K to 300 K, which reflects the typical semiconductor characteristics. Thus, the resistive switching characteristic of Al/a-SiN_x_O_y_:H/P^+^-Si device has no relation with metal conductive pathway.

**Figure 3:**
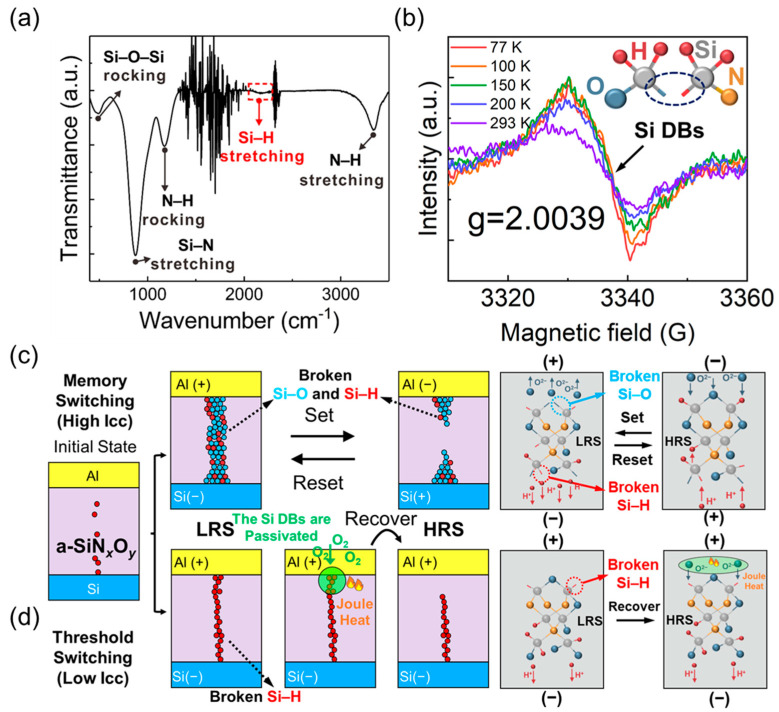
(**a**) FTIR spectrum of the as-deposited a-SiN_x_O_y_:H layer; (**b**) Temperature-dependent ESR spectra of the a-SiN_x_O_y_:H device; Schematic diagram of (**c**) memory switching and (**d**) threshold switching mechanism in Al/a–SiN_x_O_y_:H/P^+^–Si device.

To reveal the relationship between resistive switching and the atomic configurations of a-SiN_x_O_y_:H films, we analyzed the corresponding Fourier Transform infrared (FTIR) spectroscopy and electron spin resonance (ESR) spectra as shown in Figure 3a,b. The absorption bands at 841 cm^−1^, 1176 cm^−1^, 2173 cm^−1^ as well as 3356 cm^−1^, correspond to the Si–N stretching, N–H rocking, Si–H stretching, and N–H stretching modes, respectively [32]. In the case of O, as evidenced by the absorption close to the 475 cm^−1^ band, which is assigned to rocking mode of the Si–O–Si group [33]. The Si–H and N–H bonds are derived from the hydrogenation of SiN_x_O_y_. The Si–H bonds can be ascribed to the excess Si atoms combined with H atoms in the SiN_x_O_y_ films, which is confirmed by the Si^0^ peak of the XPS spectrum in Figure 1c. The area percentage of Si^0^ peak is 9.8%, which is the origin of Si–H bonds. As shown in Figure 3b, a resonance peak with a g value of 2.0039 is observed. It is related to the paramagnetic center of the Si dangling bonds (≡Si). As reported by Shamekh et al. [34], the g value of the Si dangling bond center ranges from 2.0055 to 2.0018 when Si atoms are replaced by O atoms. Our detection of g value of 2.0039 agrees with the value as reported. It indicates that Si dangling bonds are the main defect in a-SiN_x_O_y_:H films during the chemical vapor deposition. As revealed by the FTIR and ESR, Si–H bonds and a few Si dangling bonds coexist in the pristine a-SiN_x_O_y_:H films. The breakage of Si–H bonds can occur during the forming or set process due to the field-enhanced thermal breakage, because the Si–H bond energy (3.0 eV) is much lower than that of Si–O (5.4 eV) [35]. The H^+^ ions from broken Si–H bonds migrate toward the cathode under the positive electric field, producing more Si dangling bonds in the film. A schematic illustration of MS and TS is shown in Figure 3c,d.

**Figure 4:**
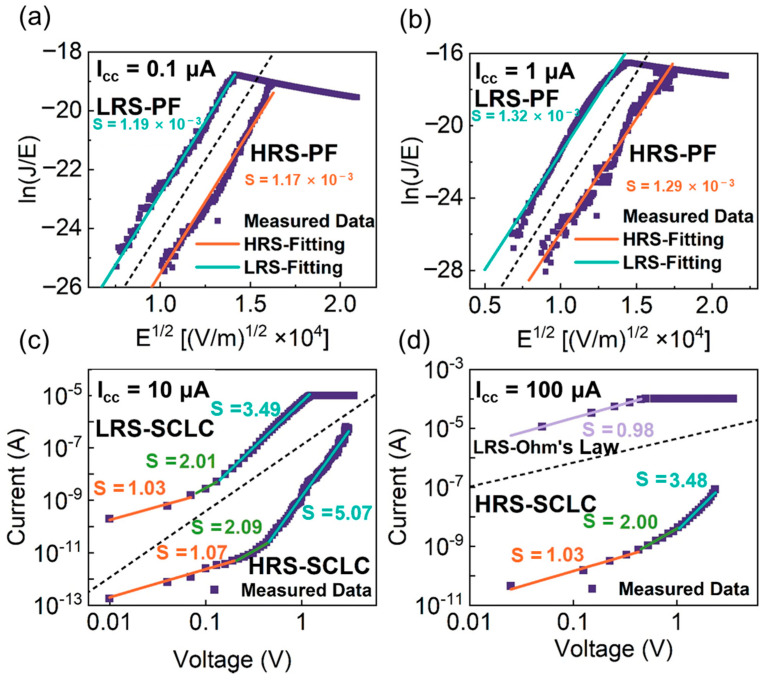
Comparison of experimental and theoretical calculation of the I–V curves for Al/a–SiN_x_O_y_:H/P^+^–Si device in HRS and LRS with I_cc_ of (**a**) 0.1 nA (**b**) 1 µA (**c**) 10 µA as well as (**d**) 100 µA based on P-F model, SCLC model and Ohm’s law.

In our device, the Si–O bonds and the Si-H bonds coexist in the a-SiN_x_O_y_:H films. Under the positive electric field with a higher I_cc_, the electric field is larger than that of lower I_cc_. Therefore, Si-H bonds and Si–O bonds will be broken in sequence with the positive voltage increasing [36,37,38,39], and the device will be switched to the LRS. Due to the contribution of the broken Si–H and Si–O bonds, a stronger and thicker conductive pathway of Si dangling bonds can be formed. Under the negative electric field, the H^+^ ions and O^2−^ ions will move back to passivate the abundant Si dangling bonds making the device switch to HRS as displayed in Figure 3c. Thus, MS can be observed from a-SiN_x_O_y_:H device under the higher I_cc_. In contrast with the higher I_cc_, only a small number of Si–H bonds can be broken to form a weak and thin conductive pathway under the positive bias with lower I_cc_ because the intensity of electric field is smaller than that of higher I_cc_. The formation of weak Si dangling bond pathway makes the device switch from HRS to LRS. The thinner the conductive pathway is, the bigger is the resistance. Due to the joule heat of current, the Si dangling bonds be oxidized by ambient oxygen [36]. And the continuous Si dangling bond pathway under the top electrode will be destroyed. Therefore, the device can spontaneously recover to HRS, which explains the reason why the TS occurs in a-SiN_x_O_y_:H device under lower I_cc_ bonds. The $\varepsilon_{r}\varepsilon_{0}$ values of 6.28 and 6.07 were obtained from the slope of P-F plots, which are lower than the static permittivity (ε = 7) [40]. The parallel fitted lines suggest that the conduction mechanism remains unchanged after resistive switching, and the smaller value of $\varepsilon_{r}$ in LRS means that a few Si dangling bonds have been formed in the dielectric layer under the electric field. The similar changing trend of I–V curves for LRS and HRS is also observed from the device with I_cc_ = 1 µA as shown in Figure 4b. The $\varepsilon_{r}$ values of 4.93 and 4.85 were obtained from the corresponding P-F plots. Compared with that of I_cc_ = 0.1 µA, the charge transportation with I_cc_ = 1 µA has the same conduction model. The size of conductive pathway can be confined with the lower I_cc_ = 1 µA, leading the formation of the weak conductive pathway. It is easier to be broken up due to the joule heat, which is the origin of the threshold switching. The various concentration of Si dangling bonds in the conductive pathway results in the different permittivity due to the various amplitude of I_cc_. When the I_cc_ increases to 10 µA, the I–V curves were plotted using a log-log scale to reveal the power law relation ($I\propto {\;V}^{m}$), as displayed in Figure 4c. The slope of I–V curve is 1.07 in the region of low voltage (<0.2 V), which obeys Ohm’s law ($I\propto {\;V}^{1}$). The thermal excitation in the conduction band is the cause of the mobile electrons. With the voltage increasing, the rupture of an Si–H bond can be realized under the electric field with H^+^ migrating toward the cathode. With the number of Si dangling bonds increasing, Si dangling bonds conductive pathway is formed, which makes the current not follow Ohm’s law anymore.

Furthermore, the migration of carriers follows the Child’s law ($I\propto {\;V}^{2}$) with an I−V slope of 2.09. Under the higher bias (V_set_), the breakage of the Si–H bonds leads to the formation of more Si dangling bonds. Once the number of Si dangling bonds arrives at the highest level, a sharp jump of current can be observed with the slope increasing from 2.09 to 5.07, making the device switch from HRS to LRS, as shown in Figure 4c. This charge-transport behavior is consistent with a space charge limited conduction (SCLC) model, and the current density J_SCLC_ can be expressed as

(1) $$J_{\mathrm{SCLC}}=\frac{9\varepsilon \mu \theta }{8d^{3}}V^{2}$$

where θ is the ratio of the free current density to the total current densities, μ is the electron mobility, ε is static dielectric constant, V is the applied voltage, and d is the film thickness [41,42]. As shown in Figure 4c, the current of HRS and LRS follows the SCLC model with the higher I_cc_ of 10 µA. Although the Si dangling bond conductive pathway becomes thicker compared with that of I_cc_ = 0.1 µA and I_cc_ = 1 µA, it is not too strong to be destroyed by the Joule heat. TS characteristic can be still observed from the device with I_cc_ = 10 µA as displayed in Figure 2c. When I_cc_ increases to 100 µA, the I–V curve of HRS follows the SCLC model, as shown in Figure 4d, but the slope of LRS remains constant, which is not in agreement of SCLC model. It is found that the I–V curve follows the Ohm’s law. It means more Si dangling bonds from the broken Si-H and Si-O bonds emerge in the resistive switching layer, which induce the formation of the extremely thicker conductive pathway. Therefore, MS characteristic can be observed with I_cc_ = 100 µA. The theoretical calculation provides important insights into the relationship between I_cc_ and conduction mechanism. As for the device with TS characteristic, the P-F emission model dominate for the carrier transportation when I_cc_ ≤ 1 µA. When I_cc_ = 10 µA, the I–V characteristics follows the SCLC model. As for the device with MS characteristic, the I–V curve of HRS and LRS follows a SCLC model and Ohm’s law, respectively. As a consequence, the evolution from TS to MS can be realized by tuning the Si dangling bond pathway, which is restricted by the compliance current. The evolution from the weak to the strong Si dangling bond conductive pathway is consistent with the change from TS to MS characteristic.

**Figure 5:**
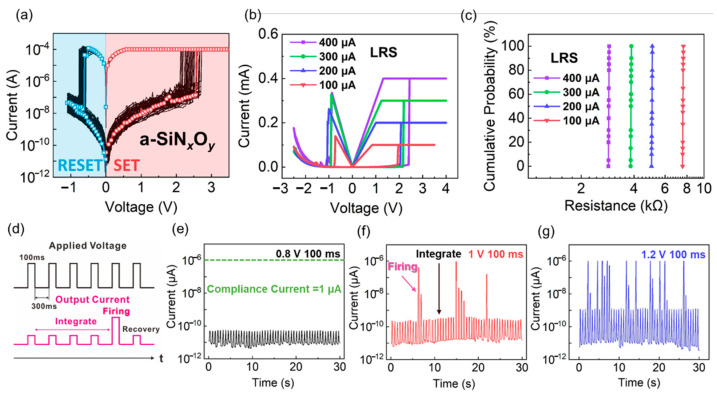
(**a**) Memory switching characteristic of Al/a-SiN_x_O_y_:H/P^+^–Si device with I_cc_ of 100 µA; (**b**) Multilevel resistive switching characteristic of Al/a-SiN_x_O_y_:H/P^+^–Si device under different I_cc_ from 100 to 400 uA; (**c**) The cumulative probability of multilevel states under different I_cc_ for 20 switching cycles; (**d**) The method and mechanism of the integrate-and-fire function of neuron tests; (**e**–**g**) The output results when the amplitude is 0.8 V, 1 V and 1.2 V, respectively. There is no firing when the amplitude of the applied pulse is 0.8 V. The firing occurs when the amplitude is 1 V. And the firing frequency increases when the amplitude of the applied pulse increases to 1.2 V.

To push the device with MS characteristic to mimic the synaptic behavior, we carried out the multilevel resistive switching investigation. Figure 5a shows bipolar MS characteristic of a-SiN_x_O_y_:H device after 50 consecutive cycles under I_cc_ of 100 µA. A stable memory windows is clearly shown, which can be ascribed to the strong and stable Si dangling bond conductive pathway. Since the evolution from TS to MS characteristic can be controlled by the tuning the number of Si dangling bond under different compliance current, the size of Si dangling bond pathway in the device with MS characteristic can be further broadened by increasing the compliance current. In other words, more different intermediate resistance states are available. As shown in Figure 5b, multilevel storage states turn out to be as expected by setting different I_cc_ levels (100, 200, 300, and 400 µA). When the compliance current ranges from 100 uA to 400 µA, the device exhibits controllable memory window. It means that tunable Si dangling bond pathway has been formed in the resistive switching layer. Figure 5c shows the cumulative probabilities of LRS under different I_cc_, which can be clearly distinguished. The larger compliance current results in a controllable space for the formation of Si dangling bonds pathway, which guarantees a sufficient margin for multilevel storage and analogue synaptic weight update.

Owing to the stable volatile switching behaviors of the device with I_cc_ = 1 µA, we have implemented the integrate-and-fire (IF) function by applying successive identical pulses to the Al/a-SiN_x_O_y_:H/P^+^-Si device, which is shown in Figure 5d. In our assumption, the H^+^ ions will be driven along the negative direction when the continuously positive pulses are applied to the device, and the Si dangling bond pathway will be formed in a gradual manner, which is similar to the influx of ions in biological neurons. When the Si dangling bond pathway is strong enough to be connected with the electrodes, the resistance will be reduced abruptly, leading to a large current spike. It indicates the device successfully enters the firing state. After the firing process, Si dangling bonds will be oxidized due to the joule heat of the higher current, which leads to the breakage of Si dangling bond conductive pathway. Therefore, the device recovers to HRS. As displayed in Figure 5e–g, the device could not fire when the amplitude and width of the voltage pulse is 0.8 V and 100 ms with an interval of 550 ms. After the voltage amplitude is increased to 1 V, the firing state is obviously realized. With the voltage amplitude increasing to 1.2 V, the firing frequency becomes higher. Therefore, the firing frequency can be influenced by the voltage amplitude of pulse.

Different from the IF function of the artificial neuron device, the information transmission is the key role of artificial synapse. It causes a change in the strength of the synaptic connection, which is defined as synaptic plasticity, as the two basic elements of synaptic plasticity, short term memory (STM) and long-term memory (LTM) reflect the capability of the information processing and memory formation in the human-brain. Paired pulse facilitation (PPF) is the most well-known STM phenomena [43], which describes the temporary enhancement of synaptic weight evoked by the two consecutive pulses.

**Figure 6:**
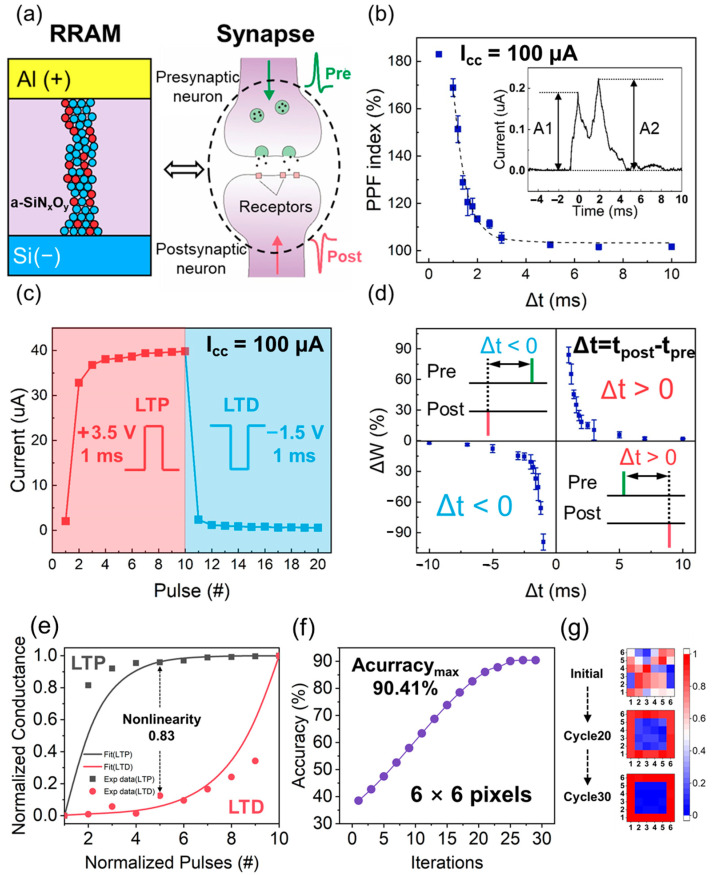
(**a**) Illustration of the structure similarity of Al/a-SiN_x_O_y_:H/P^+^-Si device to a biological synaptic junction between the pre- and postsynaptic neurons; (**b**) PPF characteristics of the electronic Al/a-SiN_x_O_y_:H/P^+^-Si synapse with I_cc_ = 100 µA; (**c**) LTP and LTD characteristic of Al/a-SiN_x_O_y_:H/P^+^-Si device with I_cc_ = 100 µA, 3.5 V/1 ms pulses is adopt for potentiating (red point) and −1.5 V/1 ms pulses is applied for depression (blue point); (**d**) STDP characteristic of the electronic Al/a-SiN_x_O_y_:H/P^+^-Si synapse with I_cc_ = 100 µA. (**e**) Normalized synaptic characteristics of a-SiN_x_O_y_:H memristor using the conventional BP-scheme (Bipolar-pulse Scheme). (**f**) The training accuracy of the neural network consisting of 6 × 6 synapses based on a-SiN_x_O_y_:H memristor. (**g**) Various images corresponding to the 6 × 6 synapses after training for 20 and 30 iterations.

Facilitation effect decreases as the interval time (Δt) between two consecutive pulse increases. As shown Figure 6a, the Al/a-SiN_x_O_y_:H/P^+^-Si device has a similar structure with a biological synapse and neuron. And the PPF index of the Al/a-SiN_x_O_y_:H/P^+^-Si device as the functions of interval time were measured using a spike amplitude of 1.5 V and a spike duration of 1 ms, as displayed in Figure 6b. In the inset, an interval time of 2 ms was selected and the corresponding PPF phenomenon is presented. A1 and A2 are the response current amplitude of the first voltage spike and the second spike, respectively. Owing to the high compliance current of 100 µA, the production of a large number of Si dangling bonds results in the formation of the thicker conductive pathway, and the device has an extremely low resistance. Therefore, the equivalent capacitance is small. When a spike is applied on the device, all the charges from the electrodes will be released immediately. Then the device will spontaneously recover to the initial state, which presents the characteristic of a short-time memory. As shown in Figure 6b, the discharging time is 2 ms. If the time interval between two consecutive spikes is smaller than 2 ms, the charges stored on the electrodes has not been released yet. The arrival of second spike will increase the charge storage, which causes the enhancement of response current, but if the interval time is larger than 2 ms, all the charges have been released before the second spike arrives. When the interval time is 1 ms, the facilitation index reaches a maximum value of 169%. When the interval time increases to 10 ms, the facilitation can be neglected. As a consequence, the index of PPF decreases exponentially as Δt increased, which is similar to the short-term memory response of biological synapses.

As the two key elements of long-term memory (LTM) [44], long-term potentiation (LTP) and long-term depression (LTD) play important roles in learning and forgetting, which can increase or decrease the synaptic weight permanently. As displayed in Figure 6c, 10 potentiation (3.5 V, 1 ms) and depression (−2.5 V, 1 ms) voltage pulses were applied on the electronic a-SiN_x_O_y_:H synapse. The conductance of a-SiN_x_O_y_:H artificial synapse increases when the continued positive spikes are applied. Conversely, the conductance of a-SiN_x_O_y_:H artificial synapse decreases distinctly as the negative spikes are applied. As for LTP, the electric field of the larger voltage spike will break Si–H and Si–O bonds in the films, and the production of abundant Si dangling bonds results in the formation of a thicker and more stable conductive pathway. The current increases abruptly after the arrival of the first pulse, which is due to high concentration of Si dangling bonds in the switching layer. In the meantime, the number of residual Si–H and Si–O bonds is reduced. Therefore, the number of new Si dangling bonds increases in a more moderate manner instead of a dramatic change under the following nine positive pulses with the same magnitude. It is observed that the stable current/conductance increases with an on/off ratio up to 19.4. As for LTD, H^+^ ions and O^2−^ ions, they will be pushed back to re-passivate a great number of Si dangling bonds under the negative pulse, which induces a dramatic decrease in current. After the arrival of the first negative pulse, the concentration of residual Si dangling bonds becomes smaller. Thus, the current also decreases moderately after the following nine negative pulses with the same magnitude, which demonstrate a long-term memory behavior.

Spike-timing-dependent plasticity (STDP) is one of the most important long-term plasticities and the prevalent weight updating rule used in the spiking neuron networks [45]. It is defined as a synaptic modification arising from the precise relative timing of fired spikes of connected neurons. The sign and magnitude of a synaptic weight depend on the relative time sequence and interval of the pre- and post-synaptic spike sequences (Δt). To demonstrate the STDP learning rule with non-overlapping spikes, presynaptic and postsynaptic spikes were applied to the electrical a-SiN_x_O_y_:H synapse as shown in Figure 6d. The pre-spike and post-spike were equal in magnitude (2 V) but opposite in voltage polarity with a duration of 1 ms. The variation in synaptic weight Δw is a function of Δt, where Δt is the difference between the timing of the presynaptic and postsynaptic spikes. It is obviously observed that the potentiation occurs when the presynaptic action potential precedes the postsynaptic firing (Δt > 0), whereas pre-synaptic activity that follows postsynaptic spike (Δt < 0) causes depression. Here, the synaptic weight change Δw was normalized as Δw = (G_s_ − G_0_)/G_0_, where G_0_ and G_s_ represent the device conductance before and after the spiking, respectively. As shown in Figure 6d, the maximum potentiation and depression value reach 84.08% and −98.22%, respectively. The relationship between the STDP and the Si dangling bond conductive pathway is analyzed as the following. Under the positive spike, Si–H and Si–O bonds can be broken to form Si dangling bonds. When the number of Si dangling bonds reach a high level, the conductive pathway is formed. It induces the enhancement of conductance, making the electrical a-SiN_x_O_y_:H synapse switch to a potentiation process. Under the negative spike, H^+^ ions and O^2−^ ions can be pushed back to passivate the Si dangling bonds, leading to the reduction in the number of Si dangling bonds. Therefore, the conductance of the device gets smaller, switching the device to a depression process. This incremental modulation of device conductance presents the capability for synaptic emulation and enables the a-SiN_x_O_y_:H film to construct neuromorphic devices for intelligence systems.

To discuss the interconnection of the training and inference, we simulated the information processing in an artificial neural network consisting of 6 × 6 synapses based on a-SiN_x_O_y_:H memristor with MS characteristic, which is used for pattern recognition. As shown in Figure 6e, nonlinearity (NL) of the weight update is defined quantitatively as:

$$\mathrm{NL}=\mathrm{Max}\left|G_{P}\left(n\right)-G_{D}\left(n\right)\right|\;\mathrm{for}\;n=1\;\mathrm{to}\;N\;$$

where G_P_(*n*) and G_D_(*n*) are the conductance values after the nth Potentiation-pulse and nth Depression-pulse, respectively. The values are normalized to the total plasticity and range from 0 to 1 during an update sequence comprising an equal number (*N*) of consecutive P-pulses and D-pulses. For a completely linear update, NL is equal to zero. Our experiment result shows that the weight update is nonlinear for both potentiation and depression in the a-SiN_x_O_y_:H memristor. The nonlinearity of the a-SiN_x_O_y_:H memristor is 0.83. Combined with the normalized conductance of potentiation and depression, the training accuracy after 30 iterations is illustrated in Figure 6f. The maximum value of accuracy reaches 90.41%, and the corresponding image of 6 × 6 pixel after training of 20 and 30 cycles is displayed in Figure 6g. It is demonstrated that the artificial synapse arrays based on a-SiN_x_O_y_:H memristor has great potential application for neuromorphic computing.

## 4. Conclusions

In summary, artificial neurons and synapses based on the Al/a-SiN_x_O_y_:H/P^+^-Si device with the tunable switching from TS to MS can be successfully obtained by controlling the compliance current. The analysis of FTIR, ESR and temperature dependent I–V characteristic reveals that volatile TS in Al/a-SiN_x_O_y_:H/P^+^-Si device under the lower compliance current is induced by the weak Si dangling bond conductive pathway, which originates from the broken Si–H bonds, while stable nonvolatile MS under the higher compliance current is attributed to the strong Si dangling bond conductive pathway, which is formed by the broken Si–H and Si–O bonds. Theoretical calculation of I–V characteristics further proves that the carrier transport of TS mainly follows the P-F model, while the charge transfer of MS agrees with the SCLC model and Ohm’s law. The controllable TS and MS characteristics of the Al/a-SiN_x_O_y_:H/P^+^-Si device can be successfully employed to mimic the biological behavior of neurons and synapses including the integrate-and-fire function, paired-pulse facilitation, long-term potentiation, long-term depression as well as spike-timing-dependent plasticity. Our discovery provides an effective way to construct the neuromorphic devices for brain-inspired computing in the AI period.

## Data Availability

The data that support the findings of this study are available from the corresponding authors upon reasonable request.
